# Effect of Divergent Feeding Regimes During Early Life on the Rumen Microbiota in Calves

**DOI:** 10.3389/fmicb.2021.711040

**Published:** 2021-10-20

**Authors:** Omar Cristobal-Carballo, Sue A. McCoard, Adrian L. Cookson, Richard A. Laven, Siva Ganesh, Sarah J. Lewis, Stefan Muetzel

**Affiliations:** ^1^Ruminant Nutrition and Physiology Team, AgResearch Grasslands, Palmerston North, New Zealand; ^2^School of Veterinary Medicine, Massey University, Palmerston North, New Zealand; ^3^Food System Integrity Team, AgResearch Grasslands, Palmerston North, New Zealand; ^4^Biostatistics Team, AgResearch Grasslands, Palmerston North, New Zealand

**Keywords:** calves, early-life, microbial community, fermentation, dietary transitions, enteric emissions

## Abstract

The objective of this study was to determine whether divergent feeding regimes during the first 41 weeks of the life of a calf are associated with long-term changes in the rumen microbiota and the associated fermentation end-products. Twenty-four calves (9 ± 5 days of age) were arranged in a 2 × 2 factorial design with two divergent treatments across three dietary phases. In phase 1 (P01), calves were offered a low-milk volume/concentrate starter diet with early weaning (CO) or high-milk volume/pasture diet and late weaning (FO). In phase 2 (P02), calves from both groups were randomly allocated to either high-quality (HQ) or low-quality (LQ) pasture grazing groups. In phase 3 (P03), calves were randomly allocated to one of two grazing groups and offered the same pasture-only diet. During each dietary phase, methane (CH_4_) and hydrogen (H_2_) emissions and dry matter intake (DMI) were measured in respiration chambers, and rumen samples for the evaluation of microbiota and short-chain fatty acid (SCFA) characterizations were collected. In P01, CO calves had a higher solid feed intake but a lower CH_4_ yield (yCH_4_) and acetate:propionate ratio (A:P) compared with FO calves. The ruminal bacterial community had lower proportions of cellulolytic bacteria in CO than FO calves. The archaeal community was dominated by *Methanobrevibacter boviskoreani* in CO calves and by *Mbb. gottschalkii* in FO calves. These differences, however, did not persist into P02. Calves offered HQ pastures had greater DMI and lower A:P ratio than calves offered LQ pastures, but yCH_4_ was similar between groups. The cellulolytic bacteria had lower proportions in HQ than LQ calves. In all groups, the archaeal community was dominated by *Mbb. gottschalkii*. No treatment interactions were observed in P02. In P03, all calves had similar DMI, CH_4_ and H_2_ emissions, SCFA proportions, and microbial compositions, and no interactions with previous treatments were observed. These results indicate that the rumen microbiota and associated fermentation end-products are driven by the diet consumed at the time of sampling and that previous dietary interventions do not lead to a detectable long-term microbial imprint or changes in rumen function.

## Introduction

The rumen is a fermentation chamber occupied by a diverse, interactive, and dynamic microbiota comprised of many species of bacteria, archaea, protozoa, and fungi (Hobson and Stewart, 1997). These microorganisms convert ingested feed into short-chain fatty acids (SCFA) and microbial biomass, which are the main source of energy and amino acids for ruminants (Puniya et al., 2015; Huws et al., 2018; Gruninger et al., 2019). Other fermentation end-products, including hydrogen (H_2_) and carbon dioxide (CO_2_), formic acid, and methyl groups, are utilized by methanogens to produce methane (CH_4_) (Moss et al., 2000; Liu and Whitman, 2008; Janssen, 2010). Methane production is both a loss of dietary gross energy (Bergman, 1990) and a greenhouse gas (Johnson and Johnson, 1995). Thus, manipulation of the ruminal ecosystem has been used in attempts to improve the efficiency of feed conversion and decrease environmental impacts (Mizrahi et al., 2021). However, manipulations in adult ruminants have shown limited and only short-term effects after treatment cessation (Weimer, 1998). The microbiota in the rumen of mature ruminants is characterized by a high degree of redundancy and resilience, which provides stability to the rumen environment and maintains the digestive function of the host across a range of feeding and management conditions (Weimer, 2015). These properties thus represent a barrier to manipulating rumen fermentation by selectively targeting groups of microorganisms. However, some studies in small ruminants suggest that in early life the rumen microbial community may be more plastic and, therefore, easier to manipulate (Yáñez-Ruiz et al., 2010; Abecia et al., 2014; De Barbieri et al., 2015).

During early postnatal life, the rumen microbiota of the young ruminant is very responsive to dietary interventions (Yáñez-Ruiz et al., 2010; Abecia et al., 2014; O’Hara et al., 2020). While sterile *in utero* (Malmuthuge and Griebel, 2018; Husso et al., 2021), the rumen of newborn animals undergoes a rapid microbial colonization during and after birth from maternal (Taschuk and Griebel, 2012; Yeoman et al., 2018) and environmental sources (Dehority and Orpin, 1997; Curtis and Sloan, 2004). After the initial colonization, microbial groups critical to the degradation of feed have been observed in the undeveloped rumen, as early as the third day of age (Fonty et al., 1987; Minato et al., 1992; Guzman et al., 2015; Wang et al., 2017). The rumen microbiota rapidly shifts toward obligate anaerobic microbes as young ruminants start to transition from milk to solid diets (Walters et al., 2011; Rey et al., 2014). In post-weaned calves, the consumption of solid diets is associated with a progressive shift in ruminal microbial composition toward a more diverse microbiota (Rey et al., 2014; Dias et al., 2017; Dill-McFarland et al., 2017). The ruminal microbiota in young ruminants acquires an adult-like composition as the solid feed intake increases between the weaning transition and 1 year of age (Dill-McFarland et al., 2019), with recent studies indicating that an increased solid feed intake can result in adult-like fermentation profiles (Cristobal-Carballo et al., 2019). As a result, dietary interventions, aimed at altering ruminal microbial composition and fermentation profiles, may be most effective during the weaning transition of young ruminants. However, there is little information available on the effect of early life nutrition of calves during the transition to weaning and immediately afterward on the rumen prokaryotic community and fermentation profiles. The aim of this study was to determine whether contrasting feeding regimes pre- and post-weaning could imprint the rumen microbial community and produce associated changes in rumen fermentation.

## Materials and Methods

Animal procedures were reviewed and approved by the Grasslands Animal Ethics Committee (AE 13297) and complied with the institutional Codes of Ethical Conduct for the Use of Animals in Research, Testing and Teaching, as prescribed in the New Zealand Animal Welfare Act of 1999 and its amendments.

### Experimental Design

Twenty-four calves were randomly selected and balanced across dietary treatments from a parent production study using 200 Hereford–Friesian-cross female calves (Burggraaf et al., 2020). Treatments in the large production study were balanced for live weight and arrival date of the calves. The study was carried out in a 2 × 2 factorial design with different dietary treatments across three dietary phases. In phase 1 (P01, 0–14 weeks), calves were reared using either a low-milk volume and concentrate starter diet with early weaning (CO) or high-milk volume and pasture diet with later weaning (FO). In phase 2, post-weaning (P02, 14–19 weeks), each group of calves was evenly divided and randomly allocated to either a high-quality (HQ) or low-quality pasture (LQ) diet. The outcome was the generation of four groups in P02, where the main effects of pre-weaning rearing system and post-weaning diet quality and the interactions were compared. In phase 3 (P03, 30 to 41 weeks), all calves were randomly allocated to two groups that equally represented all four treatment groups and managed under commercial grazing conditions on the same farm. For this study, measurements and sampling were undertaken in week 9 (P01), week 19 (P02), and week 41 (P03).

### Animal and Feeding Management

Calves from P01 were managed in two pre-weaning rearing systems: FO calves were housed during week 1 and then moved to paddocks of ryegrass/white clover pasture from weeks 2 to 12. These calves were fed whole milk powder (WMP; Table 1; NZ Agbiz, Auckland, New Zealand) at 8.0 L/calf/day (1,000 g of WMP; 125 g/L of water), divided in two feeds for 5 weeks. In this group, the intake of WMP was increased from 5.0 to 8.0 L/calf/day during the first 2 weeks. Calves were fed 8.0 L once per day from weeks 5 to 9, then 4 L once a day for 2 weeks, then were gradually weaned over the following week. Calves from the CO group were housed on arrival and fed WMP at 4.0 L/calf/day (500 g WMP/day; mixed as per the FO group), divided in two feeds for 5 weeks and then once a day for 2 weeks, before abruptly weaning off milk at the end of week 7. This group was offered *ad libitum* starter concentrate (Table 1; Denver Stock Feeds, Palmerston North, New Zealand) from weeks 1 to 7, then calves were transferred to paddocks of ryegrass/white clover pasture with starter concentrate reduced to 1.5 kg/calf/day for 2 weeks and finally to 1 kg/calf/day for two more weeks until weaning off concentrate starter at week 12.

**TABLE 1:** Chemical composition (% of dry matter) of the whole milk powder (WMP), concentrate and pastures^a^ fed to calves in phase 1 (P01), pastures of high^b^ (HQ) and low quality^c^ (LQ) in phase 2 (P02) and pastures^d^ fed to all calves in phase 3 (P03).

Feed	P01			P02		P03
	WMP	Pasture^a^	Concentrate	HQ Pasture^b^	LQ Pasture^c^	Pasture^d^
Dry matter (%)^e^	95.2	18.7	93.8	21.5	37.7	14.9
Crude protein^f^	24.1	14.8	19.8	19.8	7.3	19.2
ME (MJ/kg DM)^g^		10.5	13.8	10.9	7.6	11.2
NDF^h^	–	49.5	16.2	47.9	64.2	53
ADF^i^	–	25.4	5.9	25.2	35.7	27
Lignin^j^	–	1.8	–	3.8	4.2	2.9
Lipids^k^	28.4	1.1	2.3	2.3	1.1	2.8
Ash^l^	5.5	6.4	6.2	8.8	5.5	10.3
Soluble sugars^m^	41.5^n^	19.3	8	9.3	9	11.4
Starch^o^	–	–	36.8	–	–	–

*^a^Pasture was composed of ryegrass/white clover mixed sward. Chemical composition of pastures was scanned using near-infrared reflectance spectroscopy (NIRS; Corson et al., 1999).*

*^b^Calves were grazed in irrigated pastures. Chemical composition of pastures was scanned using near-infrared reflectance spectroscopy (NIRS; Corson et al., 1999).*

*^c^Calves were grazed in unirrigated pastures. Chemical composition of pastures was scanned using near-infrared reflectance spectroscopy (NIRS; Corson et al., 1999).*

*^d^Pasture was composed of ryegrass/white clover mixed sward. Chemical composition of pastures was scanned using near-infrared reflectance spectroscopy (NIRS; Corson et al., 1999).*

*^e^Method 945.15; AOAC, 2010.*

*^f^Method 992.15; AOAC, 2010.*

*^g^Metabolizable energy content of pellets was calculated using the equation ME = DOMD% × 0.16 (Alderman and Cottrill, 1996).*

*^h^Neutral detergent fiber; Method 7.074; AOAC, 1990.*

*^i^Acid detergent fiber; method 7.074; AOAC, 1990.*

*^j^Lignin; method 7.074; AOAC, 1990.*

*^k^Method 954.02; AOAC, 1990.*

*^l^Method 942.05; AOAC, 2012.*

*^m^Paul, A.A and Southgate, D.A. The Composition of Foods. 4th Edition, 1978.*

*^n^Lactose.*

*^o^Method 996.11; AOAC, 2010.*

In P02, calves in the HQ and LQ groups were grazed on pastures of either high quality (HQ; irrigated pasture) or low quality (LQ; non-irrigated pasture). Calves grouped in LQ were also fed grass silage to meet dry matter intake (DMI) requirements. The botanical composition of high-quality pastures was 31.0% of ryegrass, 35.5% of white clover, 7.3% of herbs, 3.4% other grasses, and 22.4% of dead material (DM basis), while the botanical composition of non-irrigated low-quality pastures was of 16.3% of ryegrass, 4.0% of white clover, 1.3% of herbs, 16.0% other grasses, and 61.4% of dead material (DM basis) (Burggraaf et al., 2020). In P03, from approximately 7 months of age, the calves were randomly allocated to one of two groups which were balanced for all four previous treatments and grazed commercially on ryegrass/white clover pasture. Fresh water was available *ad libitum* at all times.

### Enteric Emissions and Animal Performance Measurements

Enteric emissions [methane (CH_4_) and hydrogen (H_2_)] and animal performance [DMI and live weight (LW)] measurements were performed from the 24 selected calves, at weeks 9 (P01), 19 (P02), and 41 (P03). Before enteric emission measurements, calves were adapted to confinement conditions in covered yards as follows: in a group pen all together for the first 5 days and then in individual crates from days 5 to 7. Enteric emission measurements were carried out in open circuit respiratory chambers (Pinares-Patiño et al., 2012) over a 48-h period. The air flow through the chambers was set at 700, 1,000, and 1,200 L/min during the three measurement phases, respectively, to account for the increasing gas emissions as the solid feed intake of the calves increased. Calves entered the chambers in the morning (0900 h) when feed was offered: WMP (for FO calves in P01) and fresh solid feed (starter concentrate and/or pasture, depending on the phase and group). For LQ calves in P02, these animals received only low-quality grass (Table 1) during confinement and in respiration chambers; no grass silage was offered to these animals. Enteric emission measurements were paused for ∼45 min every morning to offer fresh feed and clean the chambers. During the adaptation and enteric emission measurement phases, pasture was cut daily, transported to the animal facility, and offered *ad libitum*. Water was available *ad libitum*.

Samples of concentrate and WMP were analyzed for chemical composition by wet chemistry (Hill Laboratories Ltd., Hamilton, New Zealand). Pasture samples of ryegrass/white clover-mixed sward were analyzed by near-infrared reflectance spectroscopy (NIRS; FeedTECH, Palmerston North, New Zealand). The chemical composition of the offered diets during the different rearing phases is shown in Table 1. During the gas measurement phases, DMI was calculated from the difference between the allowance and the residual feed. Milk DMI was not included in the calculation of the total DMI to estimate methane or hydrogen yield because most of the milk DM bypasses the rumen (Wise and Anderson, 1939) and, therefore, has little effect on rumen fermentation (Lane et al., 2000). Live weights were recorded a day before methane emission measurements during the morning and before feeding. Total daily production (g/day) of CH_4_ and H_2_ (pCH_4_ and pH_2_, respectively) were calculated from the enteric emission measurements (Pinares and Waghorn, 2014). Daily enteric emissions and animal performance parameters were used to calculate CH_4_ and H_2_ yield (yCH_4_ and yH_2_, respectively; g per kg DMI).

### Sampling and Fermentation Analysis of Rumen Contents

Rumen samples were collected in the morning *via* an oral stomach tube (Henderson et al., 2013) after enteric emission measurements and before feeding new milk and/or solid feed. Oral stomach tubing was performed using a stainless steel pipe (25 mm outside diameter, wall thickness 1.2 mm) measuring 520 mm in length with a “T” handle 350 mm from one end. The stainless steel pipe was used to guide the lavage tube over the back of the tongue to ensure it entered the rumen. The lavage tube (19 mm outside diameter) enabled contents to be aspirated using a 400-ml syringe from the center of the dorsal rumen. A modification of the technique was used at 9 and 19 weeks, where the stainless steel pipe was not used due to the size of the animals. Each sample of 10 to 30 ml of rumen fluid was subsampled for SCFA analysis and DNA extraction. For DNA analyses, 900 μl of each rumen fluid sample was snap frozen in a cool rack and immediately stored at −20°C until analysis. For SCFA analysis, 1.8 ml of each rumen sample was prepared as per Guyader et al. (2016). Gas chromatography was then used to analyze SCFA composition, as per Attwood et al. (1998), using a gas chromatograph (Model 6869, Hewlett-Packard, Montreal, QC, Canada) equipped with an auto-sampler, fitted with a Zebron ZB-FFAP 30.0 m × 0.53 mm I.D. × 1 μm film column (Phenomenex, Torrance, CA, United States) and a flame ionization detector set at 265°C.

### Microbial DNA Extraction, Library Preparation, and Sequencing

DNA was extracted from 200 μl of thawed and vortexed rumen fluid samples using the phenol–chloroform, bead beating with filtration kit for purification II (PCQI) (Rius et al., 2012; Henderson et al., 2013). Primers used for PCR amplification of bacterial and archaeal 16S rRNA genes are listed in Supplementary Table S1. Amplification reactions used for PCR targeting the regions of bacterial (30 cycles) and archaeal (35 cycles) 16S rRNA genes were prepared in triplicate as described by Kittelmann et al. (2013). PCR products were pooled, and the correct size products (∼500 bp) were verified by agarose gel electrophoresis and quantified by fluorescence using the Quant-iT dsDNA BR assay kit (Invitrogen, Carlsbad, CA, United States). Bacteria and archaea PCR reactions included a negative control for each separate amplification run. Negative control reactions containing no template DNA were performed alongside each PCR amplification and were included in subsequent analyses to confirm no amplification of product. Agarose gel electrophoresis was performed using 2 μl of PCR product on a 1% (w/v) agarose gel containing SYBR Safe. Each amplicon (150 ng) from the same target gene and region (i.e., all bacteria and archaea amplicons) was pooled. Pooled samples were concentrated (vacuum dried) and the final PCR product concentration was determined using Quant-iT dsDNA HS assay kit (Invitrogen, Carlsbad, CA, United States). Pools were purified using the NucleoMag NGS kit (Macherey-Nagel, Dueren, Germany). The final purification of amplicons was done using the QIAquick PCR Purification kit (Qiagen, Valencia, CA, USA) and the DNA concentration quantified using Quant-iT dsDNA HS assay kit (Invitrogen, Carlsbad, CA, United States). Both pools were diluted to 6.0 × 10^9^ copies per μl and combined at a “bacteria to archaea” ratio of 5:1 (Kittelmann et al., 2013). Pooled libraries were checked for quality control (QC) using Labchip GX Touch HT instrument (PerkinElmer, Waltham, MA, United States). Amplicons were sequenced using the Illumina MiSeq system according to the protocol of the manufacturer (Illumina, San Diego, CA, United States) at Massey Genome Service, Massey University, Palmerston North, New Zealand. The pooled library was run on one Illumina MiSeq (500 cycle V 2 kit). A control library for the run, Illumina-prepared PhiX, was loaded onto the Illumina MiSeq run at 20% volumes. Sequence reads were provided in fastq format. The sequences obtained were deposited in the European Nucleotide Archive under the accession number PRJEB37783.

### Phylogenetic Analysis of Sequencing Data

Sequencing reads were quality-filtered using the DynamicTrim function of SolexaQA (Cox et al., 2010). Reads were then processed and analyzed using the QIIME software package 1.8 (Caporaso et al., 2010). Sequencing reads were grouped into operational taxonomic units (OTUs) sharing over 97% and 99% similarity for bacteria and archaea, respectively, by using the UCLUST algorithm (Edgar, 2010). Sequences were assigned to phylogenetic kingdoms using the BLAST (version 2.4.0) algorithm (Altschul et al., 1990). Bacterial 16S rRNA genes were assigned using SILVA 123 (Henderson et al., 2019) and archaeal 16S rRNA genes using RIM-DB (Seedorf et al., 2014). QIIME-generated OTU tables were used for downstream statistical analysis.

### Statistical Analysis

Data were checked for normality using *Q*–*Q* plots alongside the Shapiro–Wilk’s *W* test. After normality assessment, univariate analyses were performed using a linear mixed effect (LME) model *via* the restricted maximum likelihood (REML) framework as implemented in the *NLME* package in R (Pinheiro et al., 2015; R Core Team, 2016). The resulting LME models were analyzed using analysis of variance (ANOVA). Predicted means from the model, together with estimates of the standard error of the mean and pairwise comparisons (Tukey’s or Benjamini–Hochberg test), were obtained and back transformed (where applicable) using the *PREDICTMEANS* package of R (Luo et al., 2014). Statistical significance was declared at a *P*-value ≤0.05.

Dietary effects were evaluated on animal performance, enteric emissions, and rumen fermentation data. In P01, dietary treatment (FO or CO) was used as a fixed effect and animal as a random effect. Data from P02 and P03 were analyzed using dietary treatments from P01 (FO and CO) and P02 (HQ and LQ) as fixed effects and animal as random effect. Live weight analysis for each feeding phase was adjusted using the initial LW as covariate in the model. The resulting LME models were analyzed using one-way ANOVA for P01 and a 2 × 2 factorial ANOVA for P02 and P03. Treatment effects were assessed and predicted means from the model, together with estimates of the standard errors of the means, were obtained and compared using Tukey’s test.

A total of 364 bacterial OTUs and 17 archaea OTUs (Supplementary Tables S2, S3) were analyzed after using a minimum average cutoff of 70 reads per sample. The alpha diversity of the bacterial and archaeal community of calves under contrasting dietary management conditions was analyzed separately using Shannon index in the *Vegan* package of R (Oksanen et al., 2017). Dietary treatment effects for the Shannon index of the microbial (bacterial and archaeal) community during P01, P02, and P03 were fitted in an LME model and analyzed using ANOVA as described for DMI, rumen fermentation, and gas emissions data. Predicted means from the models, together with estimates of the standard error of the mean (SEM), were obtained, and pairwise comparisons were done using Tukey’s test. Beta diversity of the bacteria and archaea community in each group of calves was analyzed using a partial least squares discriminant analysis (PLSDA) using the *mixOmics* package of R (Lê Cao et al., 2016). Groups of calves in the PLSDA analysis were assigned combining phase and treatments as follows: phase 1 (P01) corresponded to groups FO and CO, and phases 2 (P02) and 3 (P03) were the groups formed by the combination of dietary treatments from P01 and P02, resulting in FOHQ, FOLQ, COHQ, and COLQ. Additionally, a PLSDA was conducted for abundant microbes. Abundant microbes were defined as bacteria genera and archaea species with a relative abundance ≥0.70% and ≥1.00%, respectively. The aim was to identify whether the abundant microbiota showed a similar cluster separation pattern to that observed in the whole microbiota. Association scores for bacteria and archaea were visualized using clustered image maps (CIM) representing the first two dimensions (Henderson et al., 2015).

Univariate analyses were used to determine the effect of dietary treatments on the abundant microbial community. The abundant microbial community was defined from the OTU data table as those taxa with an overall relative abundance across phases ≥1.0 and ≥0.7% at bacteria phylum and genus level, respectively, and ≥1.0% at species level for archaea. After checking for normality, bacteria (phyla and genera) and archaea (species) community data were transformed using natural logarithm. The analysis of the abundant microbial community in each feeding phase was assessed as described for animal performance, enteric emissions, and rumen fermentation data. Predicted means from the models, together with estimates of the confident intervals (CI) with upper limit (UL) and lower limit (LL), were obtained and back transformed, and pairwise comparisons were done using the Benjamini–Hochberg test.

## Results

### Animal Performance, Enteric Emissions, and Rumen Fermentation

The effects of dietary treatments on animal performance, enteric emissions, and rumen fermentation are presented in Table 2. In P01 (week 9), CO calves had a 136% greater (*P* < 0.01) DMI than FO calves (total DMI, including milk intake, CO = 2.10 kg vs. FO = 1.84 kg; *P* < 0.01). Live weight was 8% lower (*P* = 0.02) in CO than in FO calves. Daily pCH_4_ was 25% higher in CO than FO calves but this was not significant (*P* = 0.06). Calves in the CO group had 47% lower (*P* < 0.01) yCH_4_ than FO calves. Hydrogen production and yield were not affected (*P* ≥ 0.33) by the dietary regime. Total SCFA concentrations in the rumen were 45% higher (*P* < 0.01) in CO than FO calves. Compared with FO calves, the proportion of acetate in CO calves was lower (*P* < 0.01), while the proportions of propionate and valerate were greater (*P* < 0.01). The proportions of butyrate and caproate were similar (*P* ≥ 0.11) in both groups, while both isobutyrate and isovalerate were lower (*P* < 0.01) in CO than FO calves.

**TABLE 2:** Effect of dietary treatments*^a^* on dry matter intake (DMI)*^b^*, live weight (LW)*^c^*, enteric emissions*^d^*, and fermentation profiles*^e^* in calves during three measurement phases*^f^*.

	P01				P02									P03								
	FO	CO	SEM	*P*-T1	FO	CO	SEM	HQ	LQ	SEM	*P*-T1	*P*-T2	*P*-int	FO	CO	SEM	HQ	LQ	SEM	*P*-T1	*P*-T2	*P*-int
DMI (kg/day)	0.89	2.1	0.039	< 0.01	3.50	3.31	0.202	3.98	2.83	0.202	0.52	< 0.01	0.81	4.11	3.91	0.168	4.20	3.81	0.168	0.4	0.12	0.87
LW (kg)	82.0	75.6	1.751	0.02	123.1	111.3	2.859	121.7	112.8	2.931	< 0.01	0.03	0.78	192.7	182.5	3.974	206.9	168.3	4.073	0.08	< 0.01	0.59
pCH_4_ (g/day)*^g^*	14.59	18.20	1.265	0.06	67.84	62.90	2.321	74.78	56.97	2.321	0.15	< 0.01	0.41	121.6	117.8	4.428	124.6	114.7	4.428	0.55	0.13	0.46
yCH_4_ (g/kg of DMI)*^h^*	16.21	8.66	0.690	< 0.01	20.60	19.42	1.412	18.87	21.16	1.412	0.56	0.27	0.91	29.81	30.41	0.901	29.91	30.32	0.901	0.65	0.75	0.68
pH_2_ (g/day)*^i^*	0.123	0.211	0.063	0.33	0.101	0.062	0.020	0.114	0.049	0.020	0.18	0.03	0.76	0.033	0.036	0.022	0.034	0.035	0.022	0.93	0.97	0.67
yH_2_ (g/kg)*^j^*	0.143	0.101	0.035	0.41	0.027	0.019	0.005	0.029	0.017	0.005	0.26	0.11	0.15	0.008	0.009	0.005	0.007	0.009	0.005	0.91	0.82	0.44
SCFA (mM)*^k^*	70.1	101.6	6.150	< 0.01	63.5	64.7	3.831	73.8	54.4	3.831	0.82	< 0.01	0.28	74.5	71.0	5.894	76.2	69.4	5.894	0.68	0.43	0.18
Acetate (%)	62.48	45.05	1.080	< 0.01	70.31	70.6	0.386	68.55	72.36	0.386	0.6	< 0.01	0.8	67.96	67.89	0.548	68.02	67.84	0.548	0.93	0.82	0.26
Propionate (%)	21.83	39.14	1.061	< 0.01	17.51	17.19	0.295	17.98	16.72	0.295	0.45	< 0.01	0.75	16.53	16.63	0.427	16.41	16.75	0.427	0.87	0.58	0.92
Butyrate (%)	12.04	10.99	0.801	0.37	9.19	8.95	0.255	9.41	8.73	0.255	0.52	0.07	0.71	11.71	11.64	0.276	11.77	11.58	0.276	0.87	0.65	0.14
Valerate (%)	1.35	3.53	0.179	< 0.01	0.94	1.00	0.039	1.20	0.74	0.039	0.32	< 0.01	0.32	0.99	1.02	0.038	0.99	1.02	0.038	0.49	0.67	0.43
Caproate (%)	0.45	0.68	0.097	0.11	0.29	0.34	0.027	0.28	0.35	0.027	0.17	0.07	0.13	0.12	0.13	0.008	0.13	0.11	0.008	0.43	0.17	0.72
Isobutyrate (%)	0.92	0.28	0.042	< 0.01	0.89	0.92	0.023	1.22	0.58	0.023	0.45	< 0.01	0.86	1.30	1.27	0.054	1.28	1.29	0.054	0.74	0.84	0.25
Isovalerate (%)	0.94	0.33	0.082	< 0.01	0.87	1.00	0.039	1.35	0.53	0.039	0.03	< 0.01	0.88	1.40	1.42	0.065	1.41	1.41	0.065	0.88	0.96	0.28

*Results are the means and standard error of the means (SEM), P-value for treatment effect for FO vs. CO (P01), treatment effect for HQ vs. LQ (P02) and their interactions (P-int).*

*^a^Dietary treatments corresponded to phase 1 (P01) concentrate (CO) vs. pasture (FO) diets and phase 2 (P02) high-quality (HQ) vs. low-quality (LQ) pastures, with measurements in P01 (9 weeks), P02 (19 weeks), and phase 3 (P03; 41 weeks) when all calves were offered a common pasture diet.*

*^b^DMI (kg/day) was measured in two consecutive days during gas emission measurements.*

*^c^LW (kg) was analyzed adjusting LW to initial LW.*

*^d^Methane (CH_4_) and hydrogen (H_2_) production in two consecutive days (g/day) and yield per kilogram of DMI (y; g/kg DMI) measured.*

*^e^Total concentrations (mM) and individual proportions (%) of short-chain fatty acids (SCFA).*

*^f^Dietary treatments in each phase were evaluated as follows: a one-way ANOVA in P01 (9 weeks) to analyze FO vs. CO diets and a 2 × 2 factorial ANOVA in P02 and P03 to evaluate FO vs. CO and HQ vs. LQ dietary treatment effects and their interactions.*

*^g^Methane production per animal (g of CH_4_/day).*

*^h^Methane yield (g of CH_4_/kg of DMI).*

*^i^Hydrogen production per animal (g of H_2_/day).*

*^j^Hydrogen yield (g of H_2_/kg of DMI).*

*^k^Short-chain fatty acids.*

In P02 (week 19), CO calves were 10% lighter (*P* < 0.01) than FO calves. Isovalerate proportions were higher in CO than FO calves (1.00 vs. 0.87; *P* = 0.03). No other effects from P01 dietary regimes (i.e., CO and FO) and no interactions between the dietary regimes in P01 and P02 (i.e., HQ and LQ) were observed (*P* ≥ 0.13). Dry matter intake was 41% greater in HQ than LQ calves. Live weight in HQ calves was 8% lower (*P* = 0.03) than in LQ calves. The pCH_4_ and pH_2_ were 31% (*P* < 0.01) and 133% (*P* = 0.03) greater in HQ than LQ calves, respectively. No differences between yCH_4_ and yH_2_ were observed between these groups (*P* ≥ 0.15). Total SCFA concentrations in HQ calves were 36% greater (*P* < 0.01) than in LQ calves. In HQ calves, acetate proportions were lower (*P* < 0.01), while propionate and valerate proportions were greater (*P* < 0.01) than in LQ calves. The proportions of butyrate and caproate were 20% lower and 8% greater in HQ than in LQ calves, but these were not significant (*P* = 0.07). Both isobutyrate and isovalerate proportions were greater (*P* < 0.01) in HQ than LQ calves.

In P03 (week 41), CO calves were 6% lighter than FO calves, but this was not significant (*P* = 0.08), while HQ calves were 23% heavier (*P* < 0.01) than LQ calves. No direct effect of previous dietary treatments (*P* ≥ 0.12) or their interactions (*P* ≥ 0.14) was observed on animal performance, enteric emissions, and rumen fermentation.

### Rumen Microbial Diversity

Negative control reactions containing no template for bacteria and archaea resulted in no 16S rRNA amplicons after PCR; therefore, no subsequent analysis was undertaken. After merging, filtering, and trimming, Illumina sequencing generated a total of 8,087,270 bacterial and archaeal 16S rRNA sequences from the 72 samples. The average number of sequences of bacteria and archaea was 97,286 ± 29,785 SD and 15,037 ± 2,875 SD as per sample, respectively, while the number of OTUs was 1,509 and 41 for bacteria and archaea, respectively.

**FIGURE 1:**
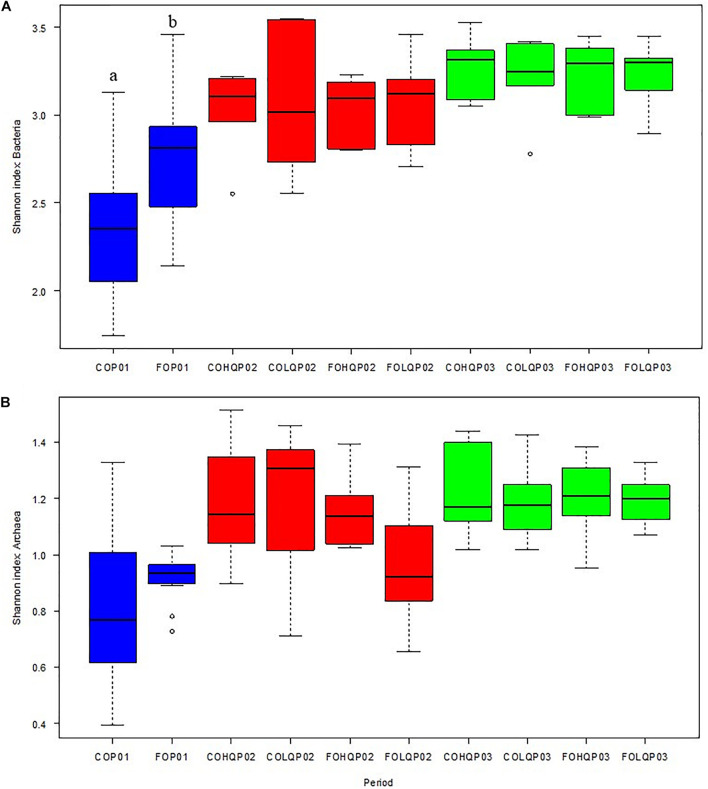
Effects of dietary treatments in the Shannon diversity indices of the microbial communities in the rumen of calves during the three measurement phases. The alpha diversity of dietary treatment concentrate (CO) vs. pasture (FO) during phase 1 (P01; blue) and the combination of dietary treatments from P01 and high-quality (HQ) vs. low-quality (LQ) pastures from phase 2 (P02), resulting in FOHQ, FOLQ, COHQ, and COLQ, evaluated during P02 (red) and phase 3 (P03; green) are shown for **(A)** bacteria Shannon index and **(B)** archaea Shannon index. Boxplots represent the 25th and 75th percentiles, the whiskers extend to the most extreme data points, lines within boxes are the medians, and dots represent outliers.

Figure 1 shows the Shannon index of the bacterial and archaeal community in each of the dietary treatment groups of calves during each sampling phase. In P01, the bacteria diversity in CO calves was lower (*P* = 0.01) than in FO calves. However, the Shannon index for bacteria in P02 and P03 did not show dietary effects from P01 and P02 (*P* ≥ 0.69) or dietary interaction effects (*P* ≥ 0.70; Figure 1A). The archaea diversity did not show dietary treatment effect (*P* ≥ 0.29) in P01, or carryover effects (*P* ≥ 0.17) from P01, dietary treatment effects during P02, or their interaction effects during P02 and P03 (Figure 1B).

**FIGURE 2:**
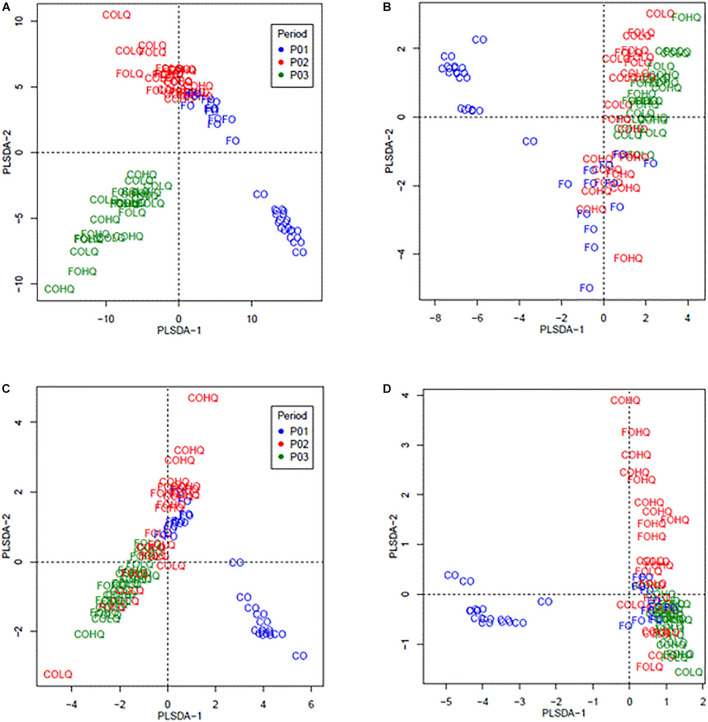
Partial least square discriminant analysis (PLSDA) of the bacteria community at the genus level and archaea community at the species level from calves fed different treatments and treatment combinations in three sampling phases. Dietary treatments corresponded to: phase 1 (P01), concentrate (CO) vs. pasture (FO); and phase 2 (P02) high-quality (HQ) vs. low-quality (LQ) pastures. The treatment groups analyzed by phase were as follows: phase 1 (P01; blue) corresponded to groups from FO and CO; phase 2 (P02; red) and phase 3 (P03; green) were the groups formed by the combination of dietary treatments from P01 and P02, resulting in FOHQ, FOLQ, COHQ, and COLQ. **(A)** PLSDA of the bacteria community—364 bacterial genera, **(B)** PLSDA of the abundant (>0.7%) bacteria—25 abundant genera, **(C)** PLSDA of the archaea community—17 archaeal species, and **(D)** PLSDA of the abundant (>1.0%) archaea—7 abundant species.

Figure 2 shows the beta diversity analysis of the bacteria and archaea for the community and abundant microbes, respectively, during each feeding phase. The PLSDA for the bacteria community (364 bacteria genus; Figure 2A) and abundant bacteria (25 genus; Figure 2B) in CO calves differed from pasture-fed calves in P01–P03. Within pasture-fed calves, the beta diversity for the bacteria community differed between calves in P01–P02 and those in P03; however, for the abundant bacteria, differences were only observed between pasture-fed calves in P01 and those in P03. The PLSDA of the archaea community (17 species; Figure 2C) and abundant archaea (7 species; Figure 2D) showed that concentrate-fed calves had different archaea diversity than those pasture-fed calves. Beta diversity within pasture-fed calves, for the archaea community and for the abundant archaea, showed that calves from the HQ groups (FOHQ and COHQ) in P02 differed from the other groups of calves in P01–P03.

### Bacterial Community

The most prominent difference in P01 was a decrease (*P* < 0.01) in the proportion of *Fibrobacteres* and *Tenericutes* in CO compared with FO calves. No other differences were observed for bacteria phyla composition between treatments (Table 3). At the genus level, members of the *Firmicutes* phylum had greater (*P* < 0.01) *Lachnospiraceae* NK3A20 group, *Roseburia*, *Erysipelotrichaceae* UCG-002, and *Succiniclasticum* proportions, but lower (*P* < 0.01) *Ruminiclostridium* 9, *Ruminococcaceae* NK4A214 group, *Ruminococcus* 1, and *Kandleria* proportions in CO compared with FO calves. On the other hand, members of the *Bacteroidetes* phylum showed greater (*P* < 0.01) *Prevotella* 7 proportions but lower (*P* < 0.01) *Prevotella* 1; *Bacteroidales* BS11, RF16, and S24-7; *Prevotellaceae* UCG-003; and *Rikenellaceae* RC9 gut group proportions in CO compared with FO calves (Table 4).

**TABLE 3:** Effect of dietary treatments*^a^* on the abundant bacteria phylum*^b^* during the measurement phases: 1 (P01), 2 (P02), and 3 (P03)*^c^*.

	**P01**										
	**CO**	**95% CI (UL–LL)**	**FO**	**95% CI (UL–LL)**	***P*-val**						

*Actinobacteria*	1.01	(0.635–1.623)	0.67	(0.420–1.073)	0.226						
*Bacteroidetes*	40.45	(33.032–49.542)	52.93	(43.219–64.821)	0.074						
*Cyanobacteria*	0.13	(0.068–0.244)	0.31	(0.164–0.591)	0.064						
*Fibrobacteres*	0.01	(0.005–0.034)	0.40	(0.158–1.017)	< 0.0001						
*Firmicutes*	48.73	(39.936–59.466)	40.45	(33.149–49.361)	0.199						
*Proteobacteria*	0.49	(0.307–0.781)	0.43	(0.267–0.679)	0.676						
*Tenericutes*	0.29	(0.176–0.486)	0.89	(0.536–1.485)	0.005						
*F:B* *^d^*	1.20	(0.786–1.846)	0.76	(0.499–1.171)	0.131						

	**P02**										
	**CO**	**95% CI (UL–LL)**	**FO**	**95% CI (UL–LL)**	**HQ**	**95% CI (UL–LL)**	**LQ**	**95% CI (UL–LL)**	***P*-T1**	***P*-T2**	***P*-int**

*Actinobacteria*	0.73	(0.547–0.986)	1.03	(0.771–1.389)	1.04	(0.774–1.395)	0.73	(0.545–0.982)	0.102	0.094	0.820
*Bacteroidetes*	55.23	(51.252–59.508)	55.46	(51.467–59.759)	53.59	(49.732–57.744)	57.15	(53.040–61.585)	0.935	0.218	0.627
*Cyanobacteria*	0.76	(0.552–1.039)	0.57	(0.412–0.776)	0.34	(0.245–0.462)	1.27	(0.927–1.746)	0.188	< 0.0001	0.576
*Fibrobacteres*	0.57	(0.377–0.851)	0.53	(0.351–0.792)	0.22	(0.144–0.324)	1.38	(0.921–2.080)	0.798	< 0.0001	0.512
*Firmicutes*	35.67	(32.431–39.233)	35.80	(32.551–39.378)	39.02	(35.480–42.921)	32.73	(29.754–35.994)	0.955	0.013	0.437
*Proteobacteria*	1.09	(0.797–1.499)	1.24	(0.901–1.695)	1.02	(0.741–1.394)	1.33	(0.969–1.822)	0.572	0.226	0.072
*Tenericutes*	1.79	(1.454–2.209)	1.49	(1.211–1.840)	1.56	(1.265–1.922)	1.72	(1.392–2.115)	0.212	0.508	0.728
*F:B* *^d^*	0.65	(0.547–0.763)	0.65	(0.546–0.763)	0.73	(0.616–0.861)	0.57	(0.485–0.677)	1.000	0.047	0.509

	**P03**										
	**CO**	**95% CI (UL–LL)**	**FO**	**95% CI (UL–LL)**	**HQ**	**95% CI (UL–LL)**	**LQ**	**95% CI (UL–LL)**	***P*-T1**	***P*-T2**	***P*-int**

*Actinobacteria*	0.6	(0.460–0.776)	0.62	(0.475–0.801)	0.71	(0.550–0.926)	0.52	(0.398–0.670)	0.858	0.082	0.338
*Bacteroidetes*	54.27	(51.198–57.522)	53.1	(50.092–56.279)	53.19	(50.178–56.376)	54.17	(51.110–57.423)	0.586	0.646	0.289
*Cyanobacteria*	0.36	(0.266–0.495)	0.32	(0.235–0.438)	0.35	(0.260–0.484)	0.33	(0.241–0.449)	0.571	0.727	0.571
*Fibrobacteres*	1.01	(0.732–1.398)	1.1	(0.795–1.518)	1.04	(0.749–1.431)	1.07	(0.776–1.483)	0.712	0.873	0.686
*Firmicutes*	36.88	(34.191–39.787)	38.57	(35.756–41.608)	37.8	(35.042–40.778)	37.63	(34.887–40.597)	0.394	0.932	0.243
*Proteobacteria*	1.19	(0.978–1.458)	1.12	(0.916–1.365)	1.31	(1.076–1.605)	1.02	(0.832–1.241)	0.631	0.072	0.914
*Tenericutes*	2.27	(1.975–2.613)	2.02	(1.756–2.324)	2.19	(1.902–2.516)	2.1	(1.824–2.413)	0.23	0.663	0.882
*F:B* *^d^*	0.68	(0.595–0.776)	0.73	(0.636–0.830)	0.72	(0.622–0.812)	0.69	(0.608–0.794)	0.469	0.803	0.259

*^a^Dietary treatments corresponded to phase 1 (P01) concentrate (CO) vs. pasture (FO) diets and phase 2 (P02) high-quality (HQ) vs. low-quality (LQ) pastures, with measurements in P01 (9 weeks), P02 (19 weeks), and phase 3 (P03; 41 weeks) when all calves were offered a common pasture diet.*

*^b^Measured effect corresponded to the seven most abundant ruminal bacterial phyla.*

*^c^Dietary treatments in each phase were evaluated as follows: a one-way ANOVA in P01 to analyze FO vs. CO diets and a 2 × 2 factorial ANOVA in P02 and P03 to evaluate FO vs. CO and HQ vs. LQ dietary treatment effects and their interactions.*

*^d^Firmicutes: Bacteroidetes ratio.*

**TABLE 4:** Effect of dietary treatments*^a^* on the abundant bacteria genus*^b^* during the three measurement phases*^c^*.

**Genus**	**P01**										

	**CO**	**95% CI (UL–LL)**	**FO**	**95% CI (UL–LL)**	***P*-val**						
		
*Bacteroidales BS11 gut group*	0.02	(0.014–0.041)	1.51	(0.875–2.592)	<0.0001						
*Bacteroidales RF16 group*	0.00	(0.002–0.004)	0.86	(0.584–1.277)	<0.0001						
*Bacteroidales S24-7 group*	0.25	(0.161–0.392)	1.48	(0.945–2.308)	<0.0001						
*Prevotella 1*	0.78	(0.321–1.893)	39.31	(16.199–95.412)	<0.0001						
*Prevotella 7*	18.38	(7.415–45.541)	0.35	(0.142–0.874)	<0.0001						
*Prevotellaceae UCG-001*	0.87	(0.443–1.701)	0.86	(0.440–1.686)	0.9852						
*Prevotellaceae UCG-003*	0.00	(0.003–0.006)	1.17	(0.758–1.808)	<0.0001						
*Rikenellaceae RC9 gut group*	0.23	(0.162–0.319)	1.66	(1.185–2.331)	<0.0001						
*Fibrobacter*	0.01	(0.005–0.036)	0.40	(0.152–1.053)	<0.0001						
*Christensenellaceae R-7 group*	0.09	(0.056–0.142)	2.94	(1.836–4.697)	<0.0001						
*Butyrivibrio 2*	0.01	(0.006–0.012)	0.41	(0.277–0.595)	<0.0001						
*Lachnospiraceae NK3A20 group*	5.95	(3.206–11.04)	1.96	(1.054–3.630)	0.0150						
*Pseudobutyrivibrio*	0.19	(0.114–0.302)	0.73	(0.451–1.196)	0.0004						
*Roseburia*	6.59	(3.899–11.127)	0.67	(0.398–1.136)	<0.0001						
*[Eubacterium] coprostanoligenes group*	0.30	(0.220–0.408)	0.94	(0.690–1.281)	<0.0001						
*Ruminiclostridium 9*	0.00	(0.003–0.008)	2.71	(1.555–4.712)	<0.0001						
*Ruminococcaceae NK4A214 group*	0.07	(0.043–0.107)	1.89	(1.204–2.970)	<0.0001						
*Ruminococcaceae UCG-014*	1.27	(0.813–1.981)	0.97	(0.624–1.520)	0.3932						
*Ruminococcus 1*	0.55	(0.308–0.985)	2.10	(1.176–3.767)	0.0027						
*Saccharofermentans*	0.00	(0.002–0.006)	0.74	(0.410–1.321)	<0.0001						
*Erysipelotrichaceae UCG-002*	3.78	(0.824–17.337)	0.09	(0.020–0.421)	0.0017						
*Kandleria*	0.00	(0.001–0.008)	1.82	(0.785–4.195)	<0.0001						
*Succiniclasticum*	2.21	(1.634–2.981)	0.95	(0.705–1.286)	0.0005						
*Selenomonas 1*	0.01	(0.007–0.020)	0.89	(0.537–1.470)	<0.0001						
*Mollicutes RF9*	0.28	(0.160–0.490)	0.80	(0.459–1.411)	0.0114						

	**P02**										
	**CO**	**95% CI (UL–LL)**	**FO**	**95% CI (UL–LL)**	**HQ**	**95% CI (UL–LL)**	**LQ**	**95% CI (UL–LL)**	***P*-T1**	***P*-T2**	***P*-int**

*Bacteroidales BS11 gut group*	2.59	(1.858–3.613)	3.28	(2.351–4.572)	1.72	(1.232–2.395)	4.9	(3.546–6.896)	0.309	0.000	0.692
*Bacteroidales RF16 group*	0.88	(0.526–1.488)	0.90	(0.538–1.522)	0.63	(0.376–1.064)	1.3	(0.752–2.129)	0.950	0.063	0.981
*Bacteroidales S24-7 group*	1.07	(0.716–1.597)	1.33	(0.892–1.989)	0.87	(0.582–1.298)	1.6	(1.098–2.447)	0.429	0.030	0.651
*Prevotella 1*	35.27	(30.002–41.453)	35.13	(29.884–41.291)	37.82	(32.174–44.455)	32.8	(27.867–38.504)	0.972	0.205	0.620
*Prevotella 7*	0.06	(0.034–0.093)	0.06	(0.039–0.106)	0.11	(0.069–0.189)	0.0	(0.019–0.053)	0.706	0.001	0.990
*Prevotellaceae UCG-001*	2.85	(1.997–4.065)	2.16	(1.512–3.078)	2.92	(2.045–4.162)	2.1	(1.477–3.007)	0.262	0.192	0.224
*Prevotellaceae UCG-003*	1.82	(1.312–2.511)	2.60	(1.883–3.602)	1.46	(1.053–2.014)	3.2	(2.347–4.491)	0.116	0.002	0.305
*Rikenellaceae RC9 gut group*	4.27	(3.432–5.324)	4.62	(3.707–5.751)	3.62	(2.904–4.505)	5.5	(4.381–6.796)	0.610	0.012	0.254
*Fibrobacter*	0.57	(0.377–0.851)	0.53	(0.351–0.792)	0.22	(0.144–0.324)	1.4	(0.921–2.080)	0.798	<0.0001	0.512
*Christensenellaceae R-7 group*	6.55	(5.607–7.656)	7.67	(6.562–8.961)	5.74	(4.913–6.709)	8.8	(7.489–10.225)	0.152	0.001	0.090
*Butyrivibrio 2*	0.92	(0.624–1.357)	0.93	(0.634–1.377)	1.41	(0.955–2.077)	0.6	(0.414–0.900)	0.955	0.005	0.636
*Lachnospiraceae NK3A20 group*	1.86	(1.449–2.382)	2.17	(1.696–2.789)	2.26	(1.763–2.897)	1.8	(1.395–2.293)	0.361	0.180	0.341
*Pseudobutyrivibrio*	1.13	(0.835–1.530)	1.05	(0.778–1.425)	1.61	(1.187–2.173)	0.7	(0.548–1.003)	0.734	0.001	0.454
*Roseburia*	0.34	(0.225–0.502)	0.27	(0.180–0.403)	0.72	(0.479–1.069)	0.1	(0.085–0.189)	0.431	<0.0001	0.561
*[Eubacterium] coprostanoligenes group*	1.07	(0.897–1.284)	1.16	(0.973–1.393)	1.00	(0.839–1.201)	1.2	(1.040–1.490)	0.511	0.093	0.036
*Ruminiclostridium 9*	0.59	(0.336–1.022)	0.62	(0.356–1.083)	1.34	(0.767–2.333)	0.3	(0.156–0.474)	0.881	0.000	0.672
*Ruminococcaceae NK4A214 group*	1.93	(1.559–2.382)	2.09	(1.691–2.585)	2.41	(1.949–2.979)	1.7	(1.352–2.067)	0.577	0.019	0.946
*Ruminococcaceae UCG-014*	1.35	(1.048–1.727)	1.15	(0.896–1.476)	1.21	(0.939–1.547)	1.3	(1.000–1.647)	0.364	0.715	0.925
*Ruminococcus 1*	1.14	(0.943–1.371)	0.99	(0.822–1.194)	0.90	(0.750–1.090)	1.2	(1.033–1.502)	0.290	0.020	0.448
*Saccharofermentans*	0.78	(0.635–0.962)	0.68	(0.548–0.831)	0.59	(0.478–0.723)	0.9	(0.730–1.105)	0.309	0.007	0.136
*Erysipelotrichaceae UCG-002*	0.01	(0.005–0.018)	0.01	(0.007–0.024)	0.02	(0.012–0.040)	0.0	(0.003–0.011)	0.565	0.006	0.377
*Kandleria*	0.09	(0.039–0.196)	0.04	(0.017–0.082)	0.42	(0.189–0.943)	0.0	(0.003–0.017)	0.127	<0.0001	0.079
*Succiniclasticum*	1.27	(0.959–1.682)	1.54	(1.159–2.034)	1.09	(0.824–1.445)	1.8	(1.350–2.367)	0.331	0.017	0.165
*Selenomonas 1*	0.00	(0.003–0.006)	0.01	(0.004–0.008)	0.01	(0.007–0.012)	0.0	(0.002–0.004)	0.269	<0.0001	0.228
*Mollicutes RF9*	0.01	(0.012–0.019)	0.01	(0.010–0.015)	0.01	(0.010–0.016)	0.0	(0.011–0.018)	0.213	0.607	0.782

	**P03**										
	**CO**	**95% CI (UL–LL)**	**FO**	**95% CI (UL–LL)**	**HQ**	**95% CI (UL–LL)**	**LQ**	**95% CI (UL–LL)**	***P*-T1**	***P*-T2**	***P*-int**

*Bacteroidales BS11 gut group*	3.66	(3.085–4.331)	3.77	(3.180–4.465)	3.71	(3.130–4.395)	3.71	(3.133–4.400)	0.795	0.993	0.198
*Bacteroidales RF16 group*	0.89	(0.644–1.218)	0.73	(0.532–1.006)	0.82	(0.594–1.123)	0.79	(0.577–1.091)	0.385	0.894	0.026
*Bacteroidales S24-7 group*	3.70	(3.034–4.510)	3.40	(2.789–4.146)	3.58	(2.937–4.366)	3.51	(2.881–4.282)	0.538	0.888	0.173
*Prevotella 1*	33.59	(29.770–37.902)	32.74	(29.019–36.945)	32.68	(28.966–36.879)	33.65	(29.824–37.971)	0.758	0.725	0.839
*Prevotella 7*	0.06	(0.040–0.093)	0.08	(0.052–0.120)	0.08	(0.050–0.116)	0.06	(0.042–0.097)	0.383	0.526	0.823
*Prevotellaceae UCG-001*	2.60	(2.148–3.141)	2.56	(2.116–3.093)	2.45	(2.028–2.965	2.71	(2.241–3.276)	0.907	0.447	0.154
*Prevotellaceae UCG-003*	2.64	(2.146–3.252)	2.42	(1.965–2.978)	2.63	(2.132–3.232)	2.43	(1.978–2.997)	0.539	0.599	0.598
*Rikenellaceae RC9 gut group*	3.71	(3.260–4.225)	3.69	(3.240–4.195)	3.64	(3.197–4.140)	3.76	(3.306–4.281)	0.937	0.706	0.754
*Fibrobacter*	1.01	(0.732–1.398)	1.10	(0.795–1.518)	1.04	(0.749–1.431)	1.07	(0.776–1.483)	0.712	0.873	0.686
*Christensenellaceae R-7 group*	7.26	(6.358–8.301)	9.17	(8.025–10.479)	8.74	(7.647–9.985)	7.62	(6.672–8.712)	0.018	0.147	0.404
*Butyrivibrio 2*	0.61	(0.466–0.796)	0.62	(0.471–0.804)	0.67	(0.509–0.869)	0.56	(0.431–0.736)	0.645	0.370	0.645
*Lachnospiraceae NK3A20 group*	0.69	(0.572–0.836)	0.86	(0.710–1.037)	0.73	(0.608–0.888)	0.81	(0.668–0.976)	0.109	0.472	0.607
*Pseudobutyrivibrio*	1.02	(0.826–1.271)	0.92	(0.738–1.136)	1.06	(0.852–1.311)	0.89	(0.715–1.101)	0.450	0.247	0.034
*Roseburia*	0.34	(0.283–0.418)	0.27	(0.222–0.327)	0.29	(0.237–0.349)	0.32	(0.266–0.392)	0.078	0.385	0.059
*[Eubacterium] coprostanoligenes group*	1.23	(1.009–1.494)	1.23	(1.013–1.501)	1.15	(0.943–1.397)	1.32	(1.084–1.605)	0.972	0.310	0.386
*Ruminiclostridium 9*	0.12	(0.066–0.204)	0.13	(0.071–0.220)	0.13	(0.075–0.234)	0.11	(0.062–0.192)	0.840	0.608	0.133
*Ruminococcaceae NK4A214 group*	2.87	(2.502–3.298)	2.98	(2.594–3.418)	3.07	(2.671–3.520)	2.79	(2.430–3.203)	0.705	0.325	0.810
*Ruminococcaceae UCG-014*	0.67	(0.531–0.843)	0.61	(0.481–0.763)	0.62	(0.492–0.781)	0.65	(0.519–0.824)	0.530	0.738	0.901
*Ruminococcus 1*	1.97	(1.519–2.552)	1.75	(1.352–2.271)	1.64	(1.263–2.121)	2.11	(1.626–2.731)	0.515	0.166	0.053
*Saccharofermentans*	1.02	(0.887–1.167)	1.00	(0.871–1.147)	0.97	(0.846–1.113)	1.05	(0.914–1.202)	0.851	0.419	0.117
*Erysipelotrichaceae UCG-002*	0.00	(0.002–0.007)	0.00	(0.001–0.003)	0.01	(0.002–0.005)	0.00	(0.001–0.005)	0.061	0.794	0.336
*Kandleria*	0.12	(0.044–0.345)	0.10	(0.035–0.277)	0.11	(0.040–0.311)	0.11	(0.039–0.307)	0.757	0.986	0.122
*Succiniclasticum*	2.66	(2.152–3.297)	2.18	(1.761–2.698)	2.20	(1.776–2.721)	2.64	(2.133–3.269)	0.181	0.219	0.053
*Selenomonas 1*	0.85	(0.545–1.335)	0.71	(0.451–1.106)	0.71	(0.456–1.118)	0.84	(0.539–1.321)	0.543	0.590	0.133
*Mollicutes RF9*	1.46	(1.182–1.793)	1.33	(1.077–1.633)	1.36	(1.106–1.677)	1.42	(1.152–1.746)	0.517	0.776	0.870

*^a^Dietary treatments corresponded to phase 1 (P01) concentrate (CO) vs. pasture (FO) diets and phase 2 (P02) high-quality (HQ) vs. low-quality (LQ) pastures, with measurements in P01 (9 weeks), P02 (19 weeks), and phase 3 (P03; 41 weeks) when all calves were offered a common pasture diet.*

*^b^Measured effect corresponded to the 35 bacterial genera with a relative abundance > 0.50% across rumen samples.*

*^c^Dietary treatments in each phase were evaluated as follows: a one-way ANOVA in P01 to analyze FO vs. CO diets and a 2 × 2 factorial ANOVA in P02 and P03 to evaluate FO vs. CO and HQ vs. LQ dietary treatment effects and their interactions.*

During P02, the bacterial community in HQ when compared with LQ calves had greater *Firmicutes* proportions (*P* < 0.01) but lower *Fibrobacteres* proportions (*P* < 0.01). At the genus level, members of the *Firmicutes* phylum in HQ calves had greater (*P* ≤ 0.02) proportions of *Butyrivibrio* 2, *Pseudobutyrivibrio*, *Roseburia*, *Ruminiclostridium* 9, and *Ruminococcaceae* NK4A214, but lower (*P* ≤ 0.02) *Christensenellaceae* R-7, *Ruminococcus* 1, *Saccharofermentans*, and *Succiniclasticum* compared with LQ calves. Conversely, members of the *Bacteroidetes* phylum in HQ calves had lower (*P* ≤ 0.03) *Bacteroidales* BS11 and S24-7, *Prevotellaceae* UCG-003, and *Rikenellaceae* RC9 gut proportions than LQ calves. No effects (*P* > 0.10) from dietary treatments in P01 or the interaction (*P* = 0.07) between P01 and P02 were observed in P02 for the most abundant bacteria phyla and genera (Tables 3, 4).

In P03, no direct effect of previous dietary treatments or their interactions was observed on the abundant bacteria at the phylum and genus levels (Tables 3, 4). Low abundant bacterial genera showed similar patterns to abundant bacteria (Supplementary Table S2).

### Archaeal Community

Table 5 shows the effects that dietary treatments during the three feeding phases have on the main archaea species in calves. During P01, the methanogenic community in CO calves was dominated by *Methanobrevibacter* (*Mbb.*) *boviskoreani*, while in FO calves, this was dominated by *Mbb*. *gottschalkii*. The abundant archaeal community in CO calves had greater (*P* < 0.01) proportions of *Mbb. boviskoreanii*, *Methanosphaera* (*Mph.*) A4, and *Mph.* Group 5, respectively, but lower (*P* < 0.01) proportions of *Mbb. gottschalkii*, *Mbb*. *ruminantium*, and *Mph. ISO3_F5* when compared with FO calves (Supplementary Figure S1).

**TABLE 5:** Effect of dietary treatments*^a^* on the abundant archaea species*^b^* during the three measurement phases*^c^*.

**Species**	**P01**										
	**CO**	**95% CI (UL–LL)**	**FO**	**95% CI (UL–LL)**	***P*-val**						

*Methanomassiliicoccales Group 10 sp.*	0.15	(0.053–0.451)	0.61	(0.210–1.789)	0.073						
*Methanobrevibacter.boviskoreani.clade*	64.03	(47.111–87.013)	0.05	(0.036–0.067)	<0.001						
*Methanobrevibacter.gottschalkii.clade*	2.53	(1.332–4.812)	57.40	(30.195–109.124)	<0.001						
*Methanobrevibacter.ruminantium.clade*	0.81	(0.272–2.416)	24.36	(8.177–72.556)	<0.001						
*Methanosphaera sp. A4*	8.58	(4.322–17.029)	0.05	(0.027–0.106)	<0.001						
*Methanosphaera sp. Group 5*	4.35	(2.719–6.954)	1.73	(1.084–2.772)	0.009						
*Methanosphaera sp. ISO3_F5*	0.07	(0.032–0.144)	4.35	(2.062–9.194)	<0.001						

	**P02**										
**Species**	**CO**	**95% CI (UL–LL)**	**FO**	**95% CI (UL–LL)**	**HQ**	**95% CI (UL–LL)**	**LQ**	**95% CI (UL–LL)**	***P*-T1**	***P*-T2**	***P*-int**

*Methanomassiliicoccales Group 10 sp.*	1.69	(0.889–3.201)	1.48	(0.777–2.799)	0.56	(0.297–1.070)	4.41	(2.325–8.372)	0.761	<0.001	0.966
*Methanobrevibacter boviskoreani.clade*	0.06	(0.023–0.149)	0.03	(0.012–0.075)	0.04	(0.014–0.088)	0.05	(0.020–0.128)	0.294	0.564	0.586
*Methanobrevibacter gottschalkii.clade*	58.91	(51.408–67.507)	65.36	(57.035–74.896)	58.16	(50.750–66.643)	66.21	(57.775–75.867)	0.274	0.176	0.787
*Methanobrevibacter.ruminantium.clade*	10.25	(5.966–17.622)	10.35	(6.020–17.782)	10.61	(6.172–18.229)	10.00	(5.820–17.191)	0.981	0.875	0.847
*Methanosphaera sp. A4*	0.08	(0.034–0.184)	0.06	(0.024–0.133)	0.13	(0.055–0.299)	0.04	(0.015–0.082)	0.577	0.036	0.327
*Methanosphaera sp. Group 5*	9.03	(5.219–15.626)	5.76	(3.327–9.960)	14.94	(8.636–25.853)	3.48	(2.011–6.020)	0.240	<0.001	0.373
*Methanosphaera sp. ISO3_F5*	3.34	(1.885–5.9226)	3.36	(1.894–5.948)	6.13	(3.460–10.870)	1.83	(1.032–3.241)	0.991	0.005	0.323

**Species**	**P03**										
	**CO**	**95% CI (UL–LL)**	**FO**	**95% CI (UL–LL)**	**HQ**	**95% CI (UL–LL)**	**LQ**	**95% CI (UL–LL)**	***P*-T1**	***P*-T2**	***P*-int**

*Methanomassiliicoccales Group 10 sp.*	4.65	(3.189–6.787)	4.60	(3.154–6.712)	3.55	(2.433–5.179)	6.03	(4.133–8.797)	0.966	0.052	0.882
*Methanobrevibacter.boviskoreani.clade*	0.04	(0.025–0.066)	0.03	(0.021–0.056)	0.05	(0.030–0.080)	0.03	(0.018–0.046)	0.628	0.113	0.016
*Methanobrevibacter.gottschalkii.clade*	64.56	(60.961–68.375)	64.22	(60.635–68.008)	62.89	(59.380–66.602)	65.93	(62.249–69.819)	0.892	0.239	0.937
*Methanobrevibacter.ruminantium.clade*	11.94	(9.892–14.405)	11.99	(9.933–14.464)	15.35	(12.719–18.521)	9.32	(7.725–11.250)	0.975	<0.001	0.833
*Methanosphaera sp. A4*	0.04	(0.009–0.133)	0.04	(0.009–0.130)	0.04	(0.010–0.143)	0.03	(0.009–0.121)	0.982	0.860	0.674
*Methanosphaera sp. Group 5*	3.01	(2.116–4.283)	2.44	(1.717–3.476)	2.40	(1.689–3.420)	3.06	(2.151–4.354)	0.393	0.324	0.200
*Methanosphaera sp. ISO3_F5*	6.92	(5.061–9.453)	10.27	(7.517–14.042)	8.93	(6.531–12.200)	7.96	(5.825–10.881)	0.077	0.595	0.668

*^a^Dietary treatments corresponded to phase 1 (P01) concentrate (CO) vs. pasture (FO) diets and phase 2 (P02) high-quality (HQ) vs. low-quality (LQ) pastures, with measurements in P01 (9 weeks), P02 (19 weeks), and phase 3 (P03; 41 weeks) when all calves were offered a common pasture diet.*

*^b^Measured effect corresponded to the seven archaeal species with a relative abundance > 1.00% across rumen samples.*

*^c^Dietary treatments in each phase were evaluated as follows: a one-way ANOVA in P01 to analyze FO vs. CO diets and a 2 × 2 factorial ANOVA in P02 and P03 to evaluate FO vs. CO and HQ vs. LQ dietary treatment effects and their interactions.*

In P02, the archaea community was dominated by *Mbb*. *gottschalkii* in both treatment groups. Compared with LQ calves, the abundant archaea community composition in HQ calves had greater (*P* ≤ 0.04) proportions of *Mph.* sp. A4, *Mph.* Group 5, and *Mph. ISO3_F5*, but lower (*P* < 0.01) proportions of *Methanomassiliicoccales* (*Mmc.*) Group 10 sp. During P02, the archaea community did not show carryover effects from P01 treatments (*P* ≥ 0.24) or interactions between P01 and P02 treatments (*P* ≥ 0.32) in the abundant archaeal species.

In P03, animals that previously grazed the HQ swards showed greater (*P* < 0.01) *Mbb. ruminantium* proportions when compared with LQ calves. No main effects of P01 diets (*P* ≥ 0.08) on the relative proportions of the abundant archaea were observed in P03.

## Discussion

Ruminal microorganisms are required for the degradation of plant components (Puniya et al., 2015; Huws et al., 2018; Gruninger et al., 2019). The establishment of these microbes in the rumen has been shown to be a dynamic progression from birth to adulthood (Jami et al., 2013; Rey et al., 2014). Recent studies have suggested that early interventions in life might imprint the microbial community, with such interventions having a persistent effect throughout the adult life of the animal (Yáñez-Ruiz et al., 2015). In this study, we have shown that feeding contrasting diets in early life (1 to 30 weeks) affects rumen fermentation patterns and rumen microbiota composition at the time of sampling; however, a permanent microbial or rumen fermentation imprint was not achieved.

### Animal Performance, Rumen Enteric Emissions, and Fermentation Profiles

In pre-weaned ruminants, the intake of solid feed is affected by milk management (e.g., amount of milk, age at weaning, and weaning method) and both access to and the type of solid feed offered (Khan et al., 2011; Abbas et al., 2017). The above was observed in the present study in P01, where pre-weaning milk management and type of solid diet access resulted in differences in solid DMI. Despite the increased DMI in CO calves, the greater daily milk allowance and duration of milk feeding in FO calves resulted in heavier pre-weaning LW, as reported in the wider cohort of animals from the parent study (Burggraaf et al., 2020) and prior studies (Muir et al., 2002; Khan et al., 2011). Differences in solid feed intake between groups corresponded to differences in CH_4_ production between CO and FO calves, where greater DMI was associated with greater CH_4_ production (Jonker et al., 2016; Bird-Gardiner et al., 2017). However, CH_4_ production per kilogram of DMI (yCH_4_) was lower in CO calves with higher energy content in grain-based diets than in FO calves with a forage diets (Table 1), as previously stated by Johnson and Johnson (1995). Differences in dietary nutrient composition and its digestibility include changes in ruminal pH and in cellulolytic activity and fiber degradation, level of starch by-pass to the intestine, and percentage of SCFA which all may influence methanogenesis (Benchaar et al., 2001; Jentsch et al., 2007). Beauchemin and McGinn (2005) indicated that feeding high-concentrate diets (47–58% of starch) decreases methane yield and lowers the acetate:propionate ratio. In the rumen, the fermentation of diets rich in structural carbohydrates produces greater proportions of acetate with the release of hydrogen, whereas the intake of diets rich in starch contents results in greater propionate proportions without hydrogen production (Ungerfeld, 2020). The propionate pathway competes for hydrogen with hydrogenotrophic methanogens (Janssen, 2010). Therefore, the high availability of starch in the diet of CO calves resulted in a reduction of methane yield in part due to an increased propionate production (Sauvant et al., 2011; Williams et al., 2019). Moreover, the rumen pH of grass-fed ruminants ranges between 6.0 and 7.0 under normal physiological conditions (Grünberg and Constable, 2009). However, the consumption of diets rich in grains results in greater concentration of SCFA and production of lactic acid that can build up in the rumen and reduce the ruminal pH below 6.0 (Dijkstra et al., 2012). Ruminal pH was not measured in the current study, but it can be speculated that CO calves with higher SCFA concentrations and less fiber contents in the diet had a lower ruminal pH than the grazing groups (Hook et al., 2011). Reduction in ruminal pH may affect methanogenic microbes and further decrease CH_4_ yield (Van Kessel and Russell, 1996).

During P02, results from the parent production trial (Burggraaf et al., 2020) showed no compensatory growth in CO calves reared on restricted milk, which correspond to observations in previous studies (Wardrop, 1966). Conversely, no growth checks were observed in FO calves, which indicated that an adequate rumen development was achieved, despite the high volume of milk fed, consistent with prior studies (Khan et al., 2011). Forage quality in this feeding phase was critical for lifetime performance of post-weaned calves, where calves fed HQ forages resulted in heavier LW when compared with calves fed LQ forages. This agrees with de Clifford et al. (2014), who showed that improved growth of calves is achieved when fed forages with higher metabolizable energy, metabolizable protein, and digestibility. The differences in methane production during P01 did not persist when these group of calves were allocated into different forage treatment diets in P02. During this P02, the intake of low-quality pastures with high fiber contents lowered DMI, resulting in low CH_4_ production (g/day). These observations corresponded to those reported in growing heifers, where DMI was reduced in forage diets with low-quality and high NDF content (Pino et al., 2018). The lack of difference in methane yield (g/kg of DM) between calves grazed in high- or low-quality pasture is likely a result of the effects that the diet had on daily methane output and DMI; this lack of a response of methane yield to pasture quality has been also shown in adult cattle (Jonker et al., 2016) and sheep (Muetzel and Clark, 2015). Conversely, the intake of grasses with high protein and low fiber content increased the total concentration of SCFA and decreased the acetate to propionate ratio, i.e., HQ calves, characteristic of the intake of grasses with high organic matter digestibility (Owens and Basalan, 2016; Pino et al., 2018). On the other hand, reduced proportions of protein degradation products, i.e., isobutyrate and isovalerate, in LQ calves reflected the low crude protein content in the pasture (Brinkhaus et al., 2017).

When the animals were on the same diet in P03, treatment differences in LW were sustained due to the lack of compensatory growth following the dietary intervention in P01 and P02. Results from the present study and the parent production study (Burggraaf et al., 2020) showed that extended nutritional restrictions imposed during the first 7 months of age limited the capacity of cattle to exhibit compensatory growth. This agrees with similar studies where severe pre- and post-weaning nutritional restrictions limit the capacity of cattle to exhibit compensatory growth and achieve equivalent weight for age in later life (Ryan, 1990; Hearnshaw, 1997; Shamay et al., 2005). No differences of DMI, CH_4_ emissions, and SCFA profiles were observed between the two groups. Similar results were observed in lambs fed high and low fiber diets in early life with no effect on rumen metabolites after 16 to 20 weeks of treatment cessation (Yáñez-Ruiz et al., 2010). Our results indicate that dietary composition at the time of measurement is the major driver for DMI, rumen fermentation pathways, and methane production independently of the previous feeding regimes, thus showing that at the metabolic level no imprint of pre- and post-weaning treatments had occurred.

### Bacterial Composition in the Rumen

The bacterial diversity in the rumen is host specific; however, variations in the composition of the ingested diet result in diversity changes of the prokaryotic domains harbored in the rumen (Henderson et al., 2015). In the present study, differences in bacterial diversity observed between treatments in the different dietary phases were consistent with those reported in young and adult ruminants, suggesting that the degradation of more structural diets is a complex process which requires a more diverse consortium of microbes working together (Kim et al., 2016; Belanche et al., 2019).

In young ruminants, the bacteria present in the rumen is largely represented by the phyla *Bacteroidetes*, *Firmicutes*, and *Proteobacteria*, whose changes in relative abundance have been associated with animal growth and diet (Li et al., 2012; Jami et al., 2013; Rey et al., 2014). Previously, reports in calves showed that the fiber content of the diet led to an increased *Firmicutes*:*Bacteroidetes* ratio (F:B ratio) (Kim et al., 2016), but no such difference was observed in P01 of the present experiment despite the differences in structural contents between diets. It cannot be elucidated whether restriction in forage intake by the allowance of high-milk volumes might affect the F:B ratio as observed in FO calves from the present study. However, in P02, the intake of low-quality pastures increased F:B ratio as previously stated. The intake of diets rich in fiber, e.g., FO in P01 and LQ in P02, resulted in increased proportion of cellulose-degrading microorganism such as *Fibrobacteres* (Ransom-Jones et al., 2012). The lack of any differences in P03 confirms that diet at the time is the major driver of the microbial community at the phylum level and that changes observed by differences in diet composition pre- and post-weaning do not lead to a permanent change of the rumen microbiota.

*Prevotella* is one of the most abundant ruminal bacterial groups and plays a key role in the degradation and utilization of a large variety of carbohydrates and proteins entering the rumen (Cotta, 1992; Kim et al., 2016; Solden et al., 2016). In the present study, *Prevotella* was the dominant genus in the rumen of calves independent of age and diet, as observed previously in young and adult ruminants (Rey et al., 2014; Henderson et al., 2015). However, our results indicate that the differences between *Prevotella* 7 and *Prevotella* 1 are driven by dietary composition. The dominance of *Prevotella* 7 was only observed in CO calves in accordance to sheep fed 95% concentrates (McLoughlin et al., 2020), while *Prevotella* 1 prevailed in FO and all the other groups of grazing calves similar to reports in sheep fed high forage diets (Xie et al., 2019). *Prevotella* 1 group includes the species *P. ruminicola*, *P. brevis*, and *P. bryantii* (Henderson et al., 2019) that produce mainly acetate and succinate (Avguštin et al., 1997), rather than propionate (Avguštin et al., 1997; Seshadri et al., 2018). *Prevotella* 1 species possess extensive repertoires of polysaccharide utilization loci and carbohydrate active enzymes targeting various plant polysaccharides (Accetto and Avguštin, 2019). *Prevotella* 7 includes species like *P. albensis* (Henderson et al., 2019) that mostly produce acetate, succinate, and propionate (Avguštin et al., 1997; Seshadri et al., 2018). Annotation of *de novo* assembled contigs from metagenomic data not only identified sequences encoding for α-amylase enzymes in uncharacterized strains of *P. albensis* but also revealed the potential to metabolize xylan as an alternative substrate (Bandarupalli and St-Pierre, 2020). The higher proportion of propionate in the rumen of concentrate-fed calves was at least partially due to the differences in these two dominant rumen bacterial genera.

The intake of concentrates in CO calves increased the relative abundance of bacteria from the genera *Roseburia*, *Lachnospiraceae* NK3A20 group, and *Erysipelotrichaceae* UCG-002, which have a high affinity for utilizing highly degradable mono- and polysaccharides (Stanton et al., 2009; Huo et al., 2014). Increases of these soluble carbohydrate-utilizing genera have been observed in the rumen contents of cattle and sheep fed greater ratios of dietary concentrates (McLoughlin et al., 2020). *Roseburia* and *Lachnospiraceae* NK3A20 are butyrate-producing microorganisms (Duncan et al., 2002). However, even though the principal fermentation product of these organisms is butyrate, no effect on the proportion or concentrations (CO = 11.3 mM vs. FO = 8.4 mM, SED = 1.62; *P* = 0.09) of this SCFA between the two groups was observed. This may be because *Roseburia* and *Lachnospiraceae* NK3A20 made up only 6.6% and 6.0% of the community, respectively. Likewise, the family *Erysipelotrichaceae* ferments a wide range of sugars to produce mainly lactic acid (Deusch et al., 2017). Studies in low methane-emitting sheep have shown that high proportions of *Erysipelotrichaceae* are associated with increases in lactic acid production, resulting in less hydrogen and methane formation (Kamke et al., 2016). In the present study, the relative abundance of *Erysipelotrichaceae* UCG-002 in the CO group may have favored the production of propionate by lactate-utilizing bacteria, i.e., *Megasphaera* (CO = 0.33% vs. FO = 0.00%, SED = 0.086; P < 0.01) (Kamke et al., 2016). Reductions in methane formation are attributed to the H_2_ utilization for propionate formation, which competes with the most common hydrogenotrophic methanogens (Liu and Whitman, 2008; Ungerfeld, 2020). Therefore, increases in the intake of starch favored the proportions of amylolytic microorganisms, whose metabolism increased the proportions of propionate during ruminal fermentation but reduced the production of hydrogen and ultimately its availability to be used by methanogens. However, although the amylolytic bacterial microbiota dominated during the pre-weaning rearing phase in concentrate-fed calves, it did not persist into the post-weaning phases of grazing calves.

Interestingly, *Kandleria*, which degrades different sugars, including D-galactose and lactose (lactate producer) (Kumar et al., 2018), was found in high proportions in calves from the FO group. The genus *Kandleria* has been isolated from the rumen of young calves fed on only milk diets (Salvetti et al., 2011). Therefore, the observed relative abundance of this genus in FO calves may be associated with the degradation of milk sugars leaking into the rumen. This can be confirmed by the low relative abundance of *Kandleria* in FO calves when transitioning into P02 and P03 despite the soluble sugars from fresh mixed sward of ryegrass and white clover.

Bacteria from the genus *Ruminococcus* and *Fibrobacter* are considered major cellulolytic degraders (Koike and Kobayashi, 2001; Ransom-Jones et al., 2012; Abdul Rahman et al., 2016). The proportion of *Fibrobacter* in P01 and P02 was higher in calves consuming diets with the highest fiber contents within each dietary phase. Such association of high relative abundance of *Fibrobacter* has been shown in heifers (Petri et al., 2013) and sheep (Belanche et al., 2019) fed forage diets. Similar effects have also been observed for the various genera in the *Ruminococcaceae*, but their proportional increases with the increase in fiber content of the diets was not as pronounced as for *Fibrobacter*, which may be due to the fact that *Ruminococcaceae* have a much wider spectrum of metabolizable substrates compared with *Fibrobacter* (Thurston et al., 1994).

Besides the known fiber degraders, some unclassified *Bacteroidetes* genera such as *Rikenellaceae* RC9, *Bacteroidales*, and *Prevotellaceae* UCG-003 and the *Firmicutes* genus *Christensenellaceae* R-7 were increased in calves fed diets rich in fiber, i.e., LQ calves. The genus *Rikenellaceae* RC9, with no as yet defined metabolic function, is one of the most prevalent microbes in the rumen microbiota (Henderson et al., 2015; De Mulder et al., 2017) and abundant in rich fibrous diets (Petri et al., 2013; Schären et al., 2017). We found similar results with increased proportions of *Rikenellaceae* in the high fiber treatments in P01 and P02. Correspondingly, hemicellulose and monomeric sugar (xylose, fucose, mannose, and rhamnose) degraders (Ormerod et al., 2016; Solden et al., 2017) from *Bacteroidales*, such as the genera BS11 gut group and S24-7 group, were increased in calves with high fiber intakes. In the present study, *Christensenellaceae* R-7 was the second most abundant bacteria genus in the rumen of grazing calves. These also appear to be related to fiber degradation as their levels were increased in the high fiber treatments in the first two phases. These findings agreed with reports in dairy cows, where increases of fiber in the diet resulted in an increase of this genus (Lima et al., 2015). Our data suggest that not only are the well-described families like *Fibrobacteracea* and *Ruminococcaceae* involved in plant fiber degradation but also members of the *Rikenellaceae* RC9, *Bacteroidales* BS11, *Bacteroidales* S24-7, and *Christensenellaceae* R-7. However, further studies of these genera are required to investigate their growth, ecology, and metabolism when ruminants are fed diets rich in fiber.

The ingestion of high-quality forage diets, i.e., HQ calves, with less NDF and ADF content favored the growth of bacteria from the *Firmicutes* genera such as *Ruminococcaceae* NK4A214, *Butyrivibrio* 2, *Ruminiclostridium* 9, and *Pseudobutyrivibrio* (Rainey, 1996; Ravachol et al., 2016). In post-weaned calves, *Butyrivibrio* and *Pseudobutyrivibrio* were found in greater proportion when transitioning into forages with lower contents of hemicellulose. Species belonging to the genus *Butyrivibrio* and *Pseudobutyrivibrio* are important degraders of plant polysaccharides, i.e., hemicelluloses (arabinoxylans) and pectin (Palevich et al., 2019b). However, some species of *Butyrivibrio* are unable to grow on structural plant components, and their role in the rumen appears to be as a utilizer of monosaccharides, disaccharides, and oligosaccharides made available by the degradative activities of other bacterial species (Palevich et al., 2017; Palevich et al., 2019a). Correspondingly, bacterial species from the genus *Pseudobutyrivibrio* are metabolically versatile and capable of growing on a range of simple mono- or oligosaccharides derived from complex plant polysaccharides such as pectins, mannans, starch, and hemicelluloses (Palevich et al., 2020). These findings may explain the increased relative abundance of *Butyrivibrio* and *Pseudobutyrivibrio* in high-quality forages.

### Archaea Composition in the Rumen

Rumen archaea are much less diverse than rumen bacteria, which likely reflects the narrow range of substrates they use (Janssen, 2010; Seedorf et al., 2014). Variations in the dietary composition have been shown to alter the archaeal community (Henderson et al., 2015) due to changes in fermentation patterns that affect the proportion of their substrates and metabolic activity (Lana et al., 1998; Ungerfeld, 2020). In the current study, the archaeal microbiota of grazing calves, across all treatments, was dominated by *Mbb. gottschalkii* and *Mbb. ruminantium*, which was in agreement with observations from adult ruminants fed diets with high fiber contents (Henderson et al., 2015; Seedorf et al., 2015). Conversely, the group of calves fed high proportions of concentrate in the diet showed increases of *Mbb. boviskoreani*, an organism that has been previously found and isolated from cattle fed diets rich in concentrates (Lee et al., 2013; Snelling et al., 2019). Therefore, these results indicate that the fermentation of diets with high contents in structural carbohydrates favored the prevalence of hydrogenotrophic archaea such as *Mbb. gottschalkii* and *Mbb. ruminantium*. Additionally, as previously discussed, rumen pH was likely to be lower in CO calves, which may decrease the relative abundance of abundant archaea species such as *Mbb. gottschalkii* and *Mbb. Ruminantium* in these calves. The metabolic activity of these methanogen species, however, begins to be inhibited when the pH drops below their optimum pH of 7.0–7.2 for growing (Miller and Lin, 2002; Janssen, 2010). In contrast, *Mbb. boviskoreani* growth is supported at a ruminal pH of 5.5, whereas its optimum pH is between 6.0 and 7.0 (Lee et al., 2013). Another observation is the low relative abundance of *Methanomassiliicoccus* (*Mmc.*) Group 10 sp. and *Methanosphaera* (*Mph.*) *ISO3_F5* in the CO group. Our results agreed with the low proportions of *Mmc.* reported in heifers fed diets rich in concentrate (Zhang et al., 2017). However, it is not clear whether the low abundance of *Mmc.* observed in concentrate-fed calves is due to a reduction in ruminal pH or a competition for substrates, e.g., methanol, with methylotrophic archaea such as *Mph*, as further discussed.

The intake of highly digestible diets, i.e., CO and HQ treatments, showed high proportions of the genera *Methanosphaera* (*Mph*). These methanogens reduce methanol (Fricke et al., 2006; Kelly et al., 2019). Methanol in the rumen is derived from the demethoxylation of dietary pectins and other methylated plant polysaccharides *via* pectin methylesterase activity (Dehority, 1969; Kelly et al., 2019). Additionally, methanol production is negatively affected by pasture maturity that has lower pectin degradation (Dehority et al., 1962). Clover and other non-grass pasture species usually contain higher proportion of pectins than grasses (Thomson, 1984; Hammond et al., 2011). In cows, the intake of diets rich in clover can favor the relative abundance of *Mph. sp. Group 5* and *Mph. sp. ISO3_F5* in the rumen (Bowen et al., 2018; Smith et al., 2020). In the present study, calves consuming high-quality sward with 35.5% of white clover (DM basis) may have produced more methanol, favoring the increase of *Mph. sp. Group 5* and *Mph. sp. ISO3_F5* when compared with the intake of non-irrigated low-quality pastures with 4.0% of white clover and more fibrous contents (Burggraaf et al., 2020). Conversely, *Mph. sp. A4* was found in high proportions in CO diets similar to that observed in pre-weaned calves by Dias et al. (2017), who indicated that the pectins present in the starter concentrate may contribute to the formation of methanol and increases in the relative abundance of this archaea species. However, further studies are required to elucidate that the production of methanol, and a hypothetical reduction in ruminal pH, in concentrate-fed calves may correspond to the increasing proportion of *Mph. sp. A4* in the rumen.

In the present study, calves consuming diets rich in fiber showed increases in *Mmc*. This order is a methylotrophic methanogen that utilizes compounds like methanol, methylamines, dimethylamine, and trimethylamine (Borrel et al., 2012; Poulsen et al., 2013). Plant-derived glycine, betaine, and choline are rapidly metabolized by ruminal bacteria using choline trimethylamine lyase (Kelly et al., 2019). Fiber-rich diets, where fermentation results in high ratios of acetate to propionate, are associated with a greater concentration of methylamine, dimethylamine, and trimethylamine compared with highly digestible diets such as corn silage (Deusch et al., 2017). In our study, the fiber content in the diets and the acetate to propionate ratios were higher in calves consuming greater fiber contents in the diet, which might have resulted in greater production of methylamines. Morgavi et al. (2015) showed that *Mph.* and *Mmc.* occupy similar trophic niches; however, the more versatile use of substrates by *Mmc.* explained their higher relative abundance in the rumen of lambs after receiving an inoculum of rumen fluid obtained from wethers fed a hay diet. Therefore, the intake of swards rich in fiber with low white clover content may produce high concentrations of methylamines in the rumen, offering a competitive advantage to low abundant methylotrophic methanogens from the order *Methanomassiliicoccales* over the genus *Methanosphaera*, whose growth is limited by the availability of methanol in the rumen (Kelly et al., 2019). Our results indicate that the apparent methanogen structure community, specifically the low abundant archaea, is affected by changes in the chemical composition of the diet consumed.

## Conclusion

In conclusion, our results showed that the rumen microbial community in the growing calf is diet dependent, with early life differences having only negligible effects on the microbiota of the growing ruminant. Different dietary regimes, pre- and post-weaning, were unable to leave a microbial imprint in the rumen of calves when the animals were fed a common diet. These findings showed that interventions after feeding colostrum to calves did not leave a permanent effect in the early microbial colonization and function in the rumen. Further studies should target earlier microbial interventions, during microbial colonization of the rumen milieu, in an attempt to imprint the ruminal microbiota.

## Data Availability Statement

The data presented in the study are deposited in the European Nucleotide Archive, accession number PRJEB37783.

## Ethics Statement

The animal study was reviewed and approved by Grasslands Animal Ethics Committee.

## Author Contributions

SMu and SMc designed the study and secured funding. OC-C, SMu, and SL generated the data. SG and OC-C completed the statistical analysis and all authors contributed to the interpretation. OC-C wrote the initial manuscript and all authors contributed to editing.

## Conflict of Interest

The authors declare that the research was conducted in the absence of any commercial or financial relationships that could be construed as a potential conflict of interest.

## Publisher’s Note

All claims expressed in this article are solely those of the authors and do not necessarily represent those of their affiliated organizations, or those of the publisher, the editors and the reviewers. Any product that may be evaluated in this article, or claim that may be made by its manufacturer, is not guaranteed or endorsed by the publisher.
