# Supplementary material for: Effect of Divergent Feeding Regimes During Early Life on the Rumen Microbiota in Calves
Source: Front Microbiol. 2021 Oct 20;12:711040. doi: 10.3389/fmicb.2021.711040 (PMC8565576; doi:10.3389/fmicb.2021.711040)

Supplementary Figure 1. Taxonomic composition of the main archaea species in calves for the dietary treatments during the three sampling phases. Dietary treatments corresponded to: phase 1 (P01), concentrate (CO) vs. pasture (FO); and phase 2 (P02) high quality (HQ) vs. low quality (LQ) pastures. The treatment groups analysed by phase were: phase one (P01) corresponded to groups from FO and CO; phase two (P02) and three (P03) were the groups formed by the combination of dietary treatments from P01 and P02, resulting in FOHQ, FOLQ, COHQ and COLQ. Columns in the charts correspond to the relative abundance (%) of the seven most abundant archaeal species within each treatment and their corresponding phases.

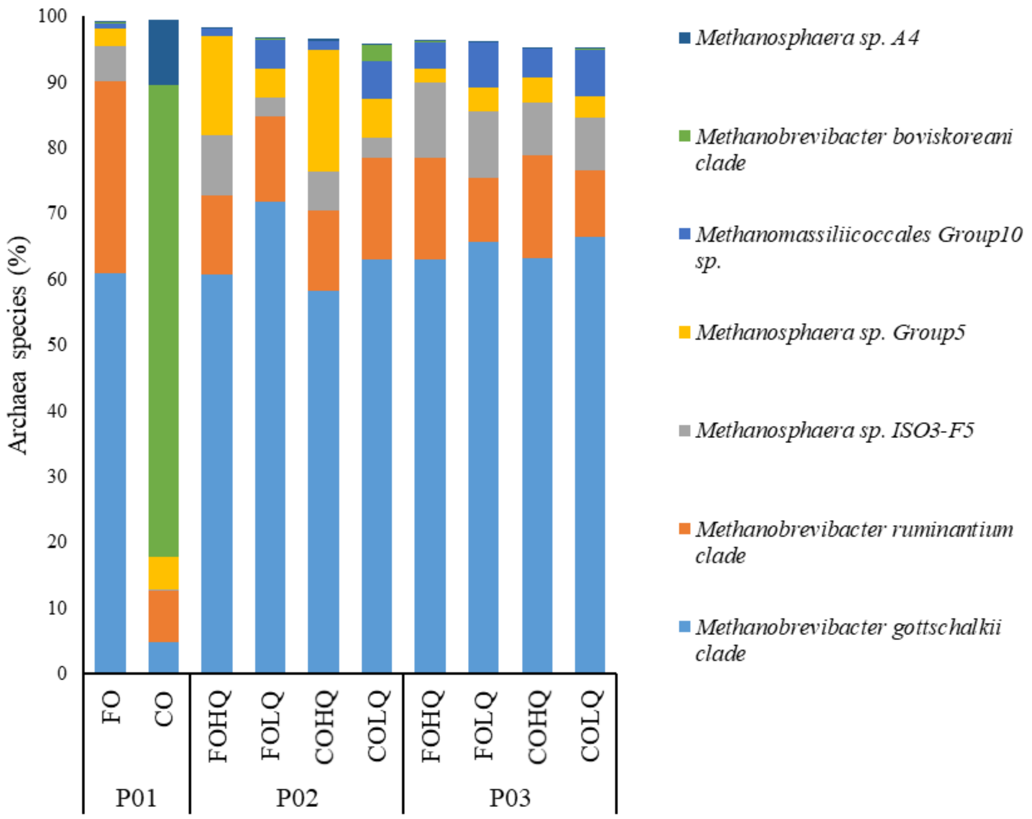

Supplement: Supplementary file 1 [file Image_1.pdf]
